# Impact of diabetes in patients waiting for invasive cardiac procedures during COVID-19 pandemic

**DOI:** 10.1186/s12933-021-01261-2

**Published:** 2021-03-23

**Authors:** Raúl Moreno, José-Luis Díez, José-Antonio Diarte, Pablo Salinas, José María de la Torre Hernández, Juan F. Andres-Cordón, Ramiro Trillo, Juan Alonso Briales, Ignacio Amat-Santos, Rafael Romaguera, José-Francisco Díaz, Beatriz Vaquerizo, Soledad Ojeda, Ignacio Cruz-González, Daniel Morena-Salas, Armando Pérez de Prado, Fernando Sarnago, Pilar Portero, Alejandro Gutierrez-Barrios, Fernando Alfonso, Eduard Bosch, Eduardo Pinar, José-Ramón Ruiz-Arroyo, Valeriano Ruiz-Quevedo, Jesús Jiménez-Mazuecos, Fernando Lozano, José-Ramón Rumoroso, Enrique Novo, Francisco J. Irazusta, Bruno García del Blanco, José Moreu, Sara M. Ballesteros-Pradas, Araceli Frutos, Manuel Villa, Eduardo Alegría-Barrero, Rosa Lázaro, Emilio Paredes

**Affiliations:** 1 University Hospital La Paz, idiPAZ, Paseo La Castellana 261, 28046 Madrid, Spain; 2 Hospital La Fe, Valencia, Spain; 3 Hospital Miguel Servet, Zaragoza, Spain; 4 Hospital Clínico San Carlos, Madrid, Spain; 5 Hospital Universitario Marqués de Valdecilla, IDIVAL, Santander, Spain; 6 Hospital German Trias I Pujol, Badalona, Spain; 7 Hospital Clínico Universitario, Santiago de Compostela, Spain; 8 Hospital Virgen de La Victoria, Málaga, Spain; 9 Hospital Clínico Universitario, Valladolid, Spain; 10 Hospital de Bellvitge, Barcelona, Spain; 11 Hospital Juan Ramón Jiménez, Huelva, Spain; 12 Hospital del Mar, Barcelona, Spain; 13 Hospital Reina Sofía, Córdoba, Spain; 14 Hospital Universitario de Salamanca, IBSAL, CIBER CV, Salamanca, Spain; 15 Hospital de Cabueñes, Gijón, Spain; 16 Complejo Hospitalario, León, Spain; 17 Hospital Doce de Octubre, Madrid, Spain; 18 Hospital San Pedro de La Rioja, Logroño, Spain; 19 Hospital Puerta del Mar, Cádiz, Spain; 20 Hospital de La Princesa, Madrid, Spain; 21 Corporació Sanitaria Parc Tauli, Sabadell, Spain; 22 Hospital Virgen de L´Arrixaca, Murcia, Spain; 23 Hospital Lozano Blesa, Zaragoza, Spain; 24 Hospital Clínico de Navarra, Pamplona, Spain; 25 Hospital Universitario, Albacete, Spain; 26 Hospital General Universitario, Ciudad Real, Spain; 27 Hospital de Galdakao, Bilbao, Spain; 28 Hospital Universitario, Guadalajara, Spain; 29 Policlínica de Guipúzcoa, San Sebastian, Spain; 30 Hospital Vall D´Hebron, Barcelona, Spain; 31 Hospital Virgen de La Salud, Toledo, Spain; 32 Hospital de Valme, Sevilla, Spain; 33 Hospital San Juan, Alicante, Spain; 34 Hospital Virgen del Rocío, Sevilla, Spain; 35 Hospital Universitario de Torrejón, Universidad Francisco Vitoria, Torrejón de Ardoz, Spain; 36 Hospital Ruber Internacional, Madrid, Spain; 37 Hospital de Torrecárdenas, Almería, Spain; 38 pInvestiga, Moaña, Pontevedra, Spain

**Keywords:** Diabetes, Interventional cardiology, COVID-19, Mortality, Waiting list

## Abstract

**Background:**

During COVID-19 pandemic, elective invasive cardiac procedures (ICP) have been frequently cancelled or postponed. Consequences may be more evident in patients with diabetes.

**Objectives:**

The objective was to identify the peculiarities of patients with DM among those in whom ICP were cancelled or postponed due to the COVID-19 pandemic, as well as to identify subgroups in which the influence of DM has higher impact on the clinical outcome.

**Methods:**

We included 2,158 patients in whom an elective ICP was cancelled or postponed during COVID-19 pandemic in 37 hospitals in Spain. Among them, 700 (32.4%) were diabetics. Patients with and without diabetes were compared.

**Results:**

Patients with diabetes were older and had a higher prevalence of other cardiovascular risk factors, previous cardiovascular history and co-morbidities. Diabetics had a higher mortality (3.0% vs. 1.0%; p = 0.001) and cardiovascular mortality (1.9% vs. 0.4%; p = 0.001). Differences were especially important in patients with valvular heart disease (mortality 6.9% vs 1.7% [p < 0.001] and cardiovascular mortality 4.9% vs 0.9% [p = 0.002] in patients with and without diabetes, respectively). In the multivariable analysis, diabetes remained as an independent risk factor both for overall and cardiovascular mortality. No significant interaction was found with other clinical variables.

**Conclusion:**

Among patients in whom an elective invasive cardiac procedure is cancelled or postponed during COVID-19 pandemic, mortality and cardiovascular mortality is higher in patients with diabetes, irrespectively on other clinical conditions. These procedures should not be cancelled in patients with diabetes.

## Introduction

The ongoing COVID-19 pandemic, caused by the severe acute respiratory syndrome coronavirus 2 (SARS-CoV-2), was declared by the World Health Organization (WHO) in March 2020 [1]. By January 2021, almost 100 million people had suffered the disease, and nearly 2 million have died worldwide. Apart from these direct consequences, health care systems have been severely overwhelmed, negatively impacting on the management of other patients that usually require prompt treatment, especially those with cardiovascular diseases [2–8]. Specifically, invasive cardiac procedures (ICP) have been cancelled or postponed in many centers, and this may have fatal consequences for some patients, as we have recently shown in a multicenter study from Spain [9].

Cardiovascular diseases constitute the main cause of death in patients with diabetes mellitus (DM) [10], and among patients with cardiovascular diseases, those with DM are at an especially high risk of death [11, 12]. Because of that, waiting list in patients pending on cardiovascular procedures that have been postponed due to the pandemic may have especial impact among diabetics [9].

The objective was to identify the peculiarities of patients with DM among those in whom ICP were cancelled or postponed due to the COVID-19 pandemic, as well as to identify subgroups in which the influence of DM has higher impact on the clinical outcome.

## Methods

### Study population

We have previously published the outcome of patients in whom elective ICP were cancelled or postponed when the state of alarm due to the COVID-19 pandemic was declared in Spain on the 14th of March 2020. In this study, 2,158 patients were included in 37 hospitals [8].

At the time of the publication of that study, DM status was known in 2,110, whereas no information was available for 48 patients. For the present sub-study, doing additional efforts, directly contacting with the patient or obtaining documents from the referral centers, we could obtain DM status for all 2158 patients.

### Data collection and follow-up

Data were entered in an electronic database (pInvestiga, Moaña, Pontevedra, Spain). Clinical variables, such as main cardiovascular disease pending on treatment, type of procedure pending to be performed, clinical situation, and cardiovascular risk factors, were collected.

Patients were followed-up until the 30th of April 2020 (45 days) [8]. Patients with DM were compared with those without DM, regarding type of pending procedure, main cardiovascular disease, functional class for heart failure and angina, and other clinical variables. The influence of DM in different patient subgroups was evaluated.

### Definitions

Coronary angiography and/or PCI as the type of pending procedure included coronary angiography in patients with previously diagnosed or suspected CAD, and those with previously known CAD pending on PCI (e.g. stage procedures). Coronary angiography to rule out CAD as the underlying cause of left ventricular dysfunction was also included in this category. However, coronary angiography as part of the study of patients pending on any type of surgical intervention was included in a different category, because the main underlying condition was considered to be the disorder pending to be treated (e.g. valvular heart disease) rather than the eventual bystander of coronary artery disease. Other procedures included transcatheter aortic valve implantation (TAVI), percutaneous mitral valve repair, left atrial appendage closure, percutaneous closure of ASD, or treatment of tricuspid regurgitation.

### Statistical analysis

Continuous variables are presented as mean ± standard deviation and compared using the Student t-test or appropriate non-parametric tests. Discrete variables are presented as percentages (proportions), and compared with the Chi-square test, using Fisher correction when needed. Statistical analysis was done using the SPSS statistical package (Chicago, Illinois). Associations were considered statistically significant when p < 0.05, although all p values are presented. Univariable and multivariable analysis were conducted in order to identify independent risk factors for mortality, and for secondary endpoints.

## Results

### Type of pending procedure

**Fig. 1:**
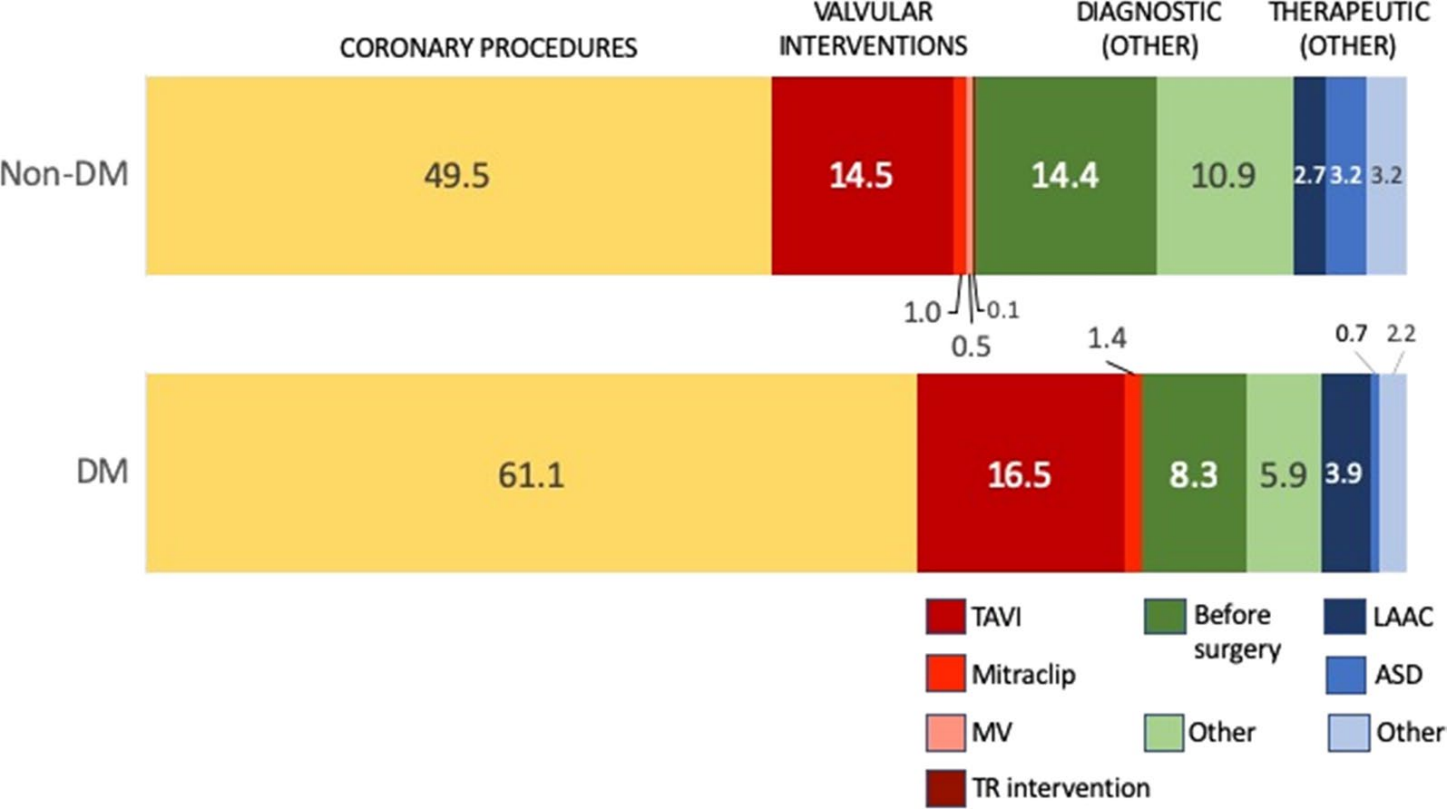
Type of pending procedures in patiens with and without DM

Out of the 2,158 patients, 700 (32.4%) were DM. Figure 1 shows the type of pending procedure in patients with and without DM. Coronary diagnostic and/or therapeutic intervention was the most frequent pending procedure for both groups of patients, but it was more frequent in DM than in non-DM (61.5% vs. 49.5%, respectively; p < 0.001). The second type of procedure was percutaneous valvular intervention, that accounted for 17.9% and 16.2% for DM and non-DM, respectively (p = 0.300). Other diagnostic procedure was pending in 14.2% and 25.2% of DM and non-DM patients, respectively (p < 0.001). Other therapeutic procedure was pending in 6.7% and 9.1% in patients with and without DM, respectively (p = 0.060).

### Comparison of patients with and without DM

Table 1 shows the differences among patients with and without DM in relation with clinical characteristics. Patients with DM were older, and had a higher prevalence of hypertension, hypercholesterolemia, chronic renal failure, peripheral artery disease, and previously diagnosed coronary artery disease including previous infarction, and previous myocardial revascularization. Left ventricular dysfunction was more frequently present in patients with DM. Functional class for heart failure (NYHA) was similar for patients with and without DM, but functional class for angina (CCS) was worse in patients with DM.

**Table 1:** Clinical characteristics of patients with and without DM

	DM		
	Yes (n = 700)	No (n = 1458)	P value
Age	72.3 ± 10.0	69.2 ± 12.7	< 0.001
Age ≥ 80 (%)	24.9	21.4	0.078
Female gender (%)	34.9	39.9	0.066
Hypertension (%)	82.9	59.8	< 0.001
Hypercholesterolemia (%)	71.0	47.9	< 0.001
Smoking (%)	30.8	29.3	0.483
Chronic renal failure (%)	13.2	7.5	< 0.001
Peripheral artery disease (%)	14.2	9.0	< 0.001
Previous CAD (%)	41.7	27.4	< 0.001
Previous infarction (%)	17.9	11.4	< 0.001
Previous PCI (%)	23.6	15.5	< 0.001
Previous CABG (%)	6.7	3.4	0.001
Previous valve replacement (%)	3.5	4.9	0.144
Left ventricular dysfunction (%)	27.1	21.4	0.033
NYHA > II (%)	20.1	19.0	0.282
CCS > II (%)	10.0	8.4	0.001
Main cardiovascular condition (%)			
Ischemic heart disease	57.6	47.0	< 0.001
Valvular heart disease	29.2	36.9	
Other	13.3	16.2	

*CAD* coronary artery disease, *PCI* Percutaneous coronary intervention, *CABG* Coronary artery bypass grafting, *NYHA* New York Heart Association, *CCS* Cardiology Canadian Society

Among the 2,158 patients, 559 had previously documented coronary artery disease. Patients with DM had higher frequency of multi-vessel disease (72.9% vs. 56.7% in non-DM, p < 0.001). Left main disease was also more frequent in patients with DM, but differences were not statistically significant (13.9% vs. 9.8% in non-DM, p = 0.133).

### Influence of DM on clinical outcomes

During the 45-day follow-up period, 36 patients died (1.7%), most of them due to cardiovascular causes (n = 19, 59.4%). Patients with DM had a higher rate of both overall mortality (3.0% vs. 1.0% in non-DM, p = 0.001) and cardiovascular mortality (1.9% vs. 0.4% in non-DM, p = 0.001), whereas differences in non-cardiovascular mortality were not statistically different (1.1% vs. 0.6% in DM and non-DM, respectively; p = 0.196).

**Fig. 2:**
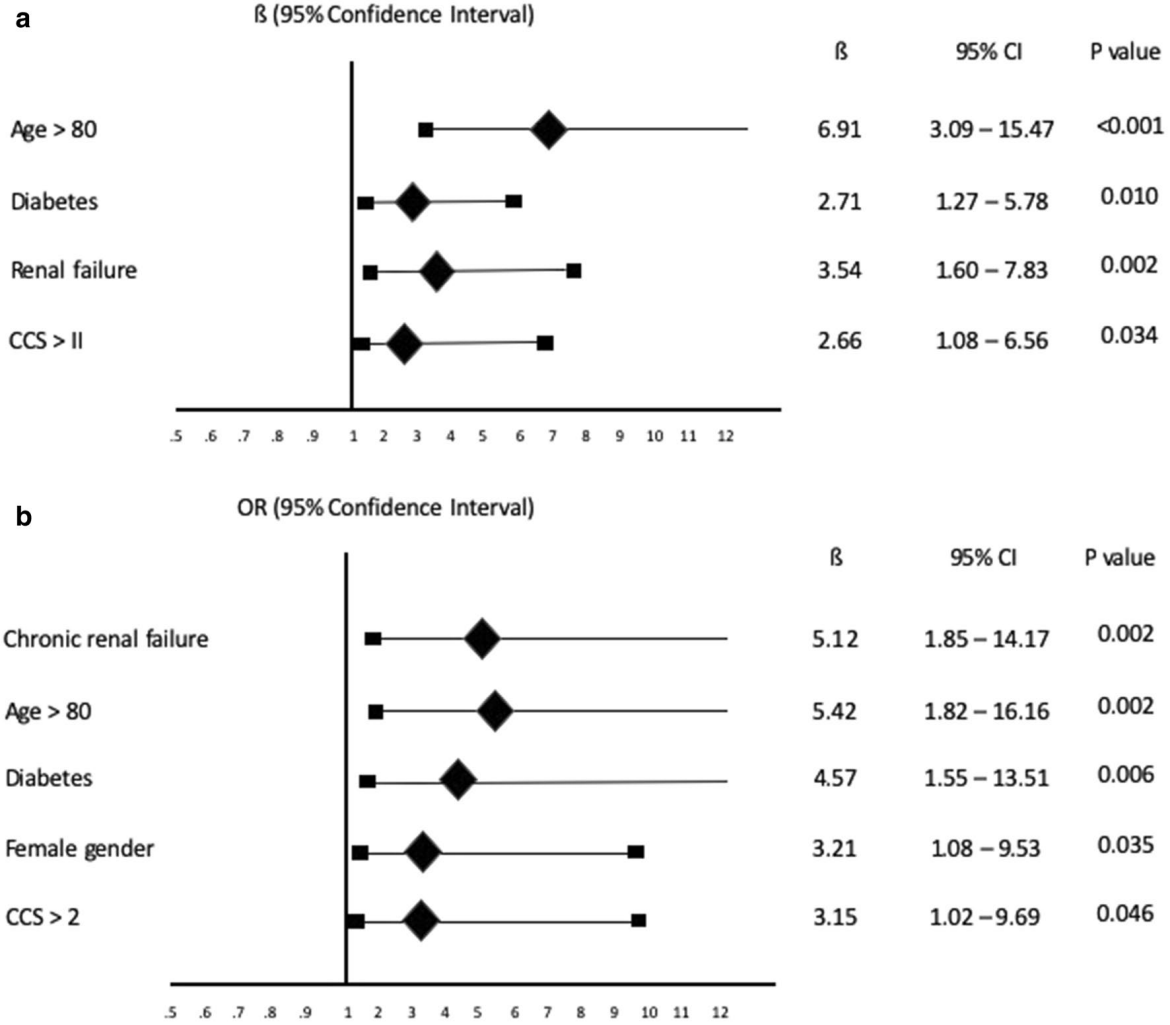
Independent risk factors for mortality (**a**) and cardiovascular mortality (**b**)

In the multivariable analysis, DM remained as an independent risk factor both for overall and cardiovascular mortality (Fig. 2).

The proportion of patients that underwent an urgent procedure due to clinical instabilization was also significantly higher in patients with DM (10.4% vs. 7.3% in non-DM; p = 0.015).

During the study period, 17 patients with DM (2.4%) and 21 without DM (1.4%) had a diagnosis of COVID-19 by protein-chain reaction test for SARS-CoV-2 (p = 0.157). Out of these patients, 11 (29.7%) died. Among patients with COVID-19 disease, 16 were diabetics, and 21 non diabetics, mortality being higher in diabetics (6/16; 37.5%) than in non diabetics (5/21; 23.8%), but difference was not statistically significant (p = 0.475).

### Mortality in patients with and without DM among different subgroups

**Fig. 3:**
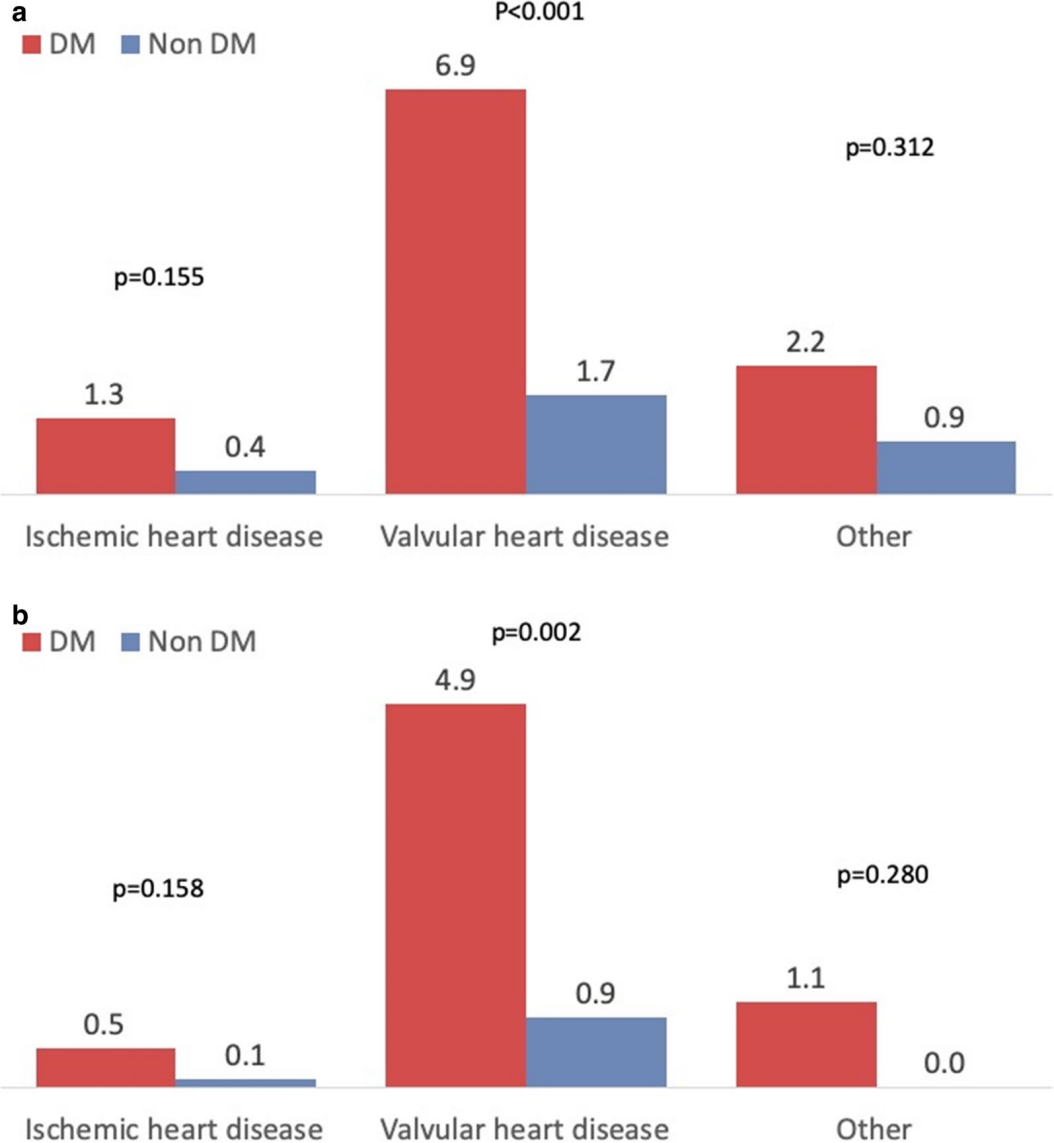
Mortality (**a**) and cardiovascular mortality (**b**) rates in patients with and without DM accordingly to the main cardiovascular disease

Mortality and cardiovascular mortality were higher in patients with DM regardless the main cardiovascular disease, although differences were statistically significant only for those patients with valvular heart disease (Fig. 3a and b).

**Fig. 4:**
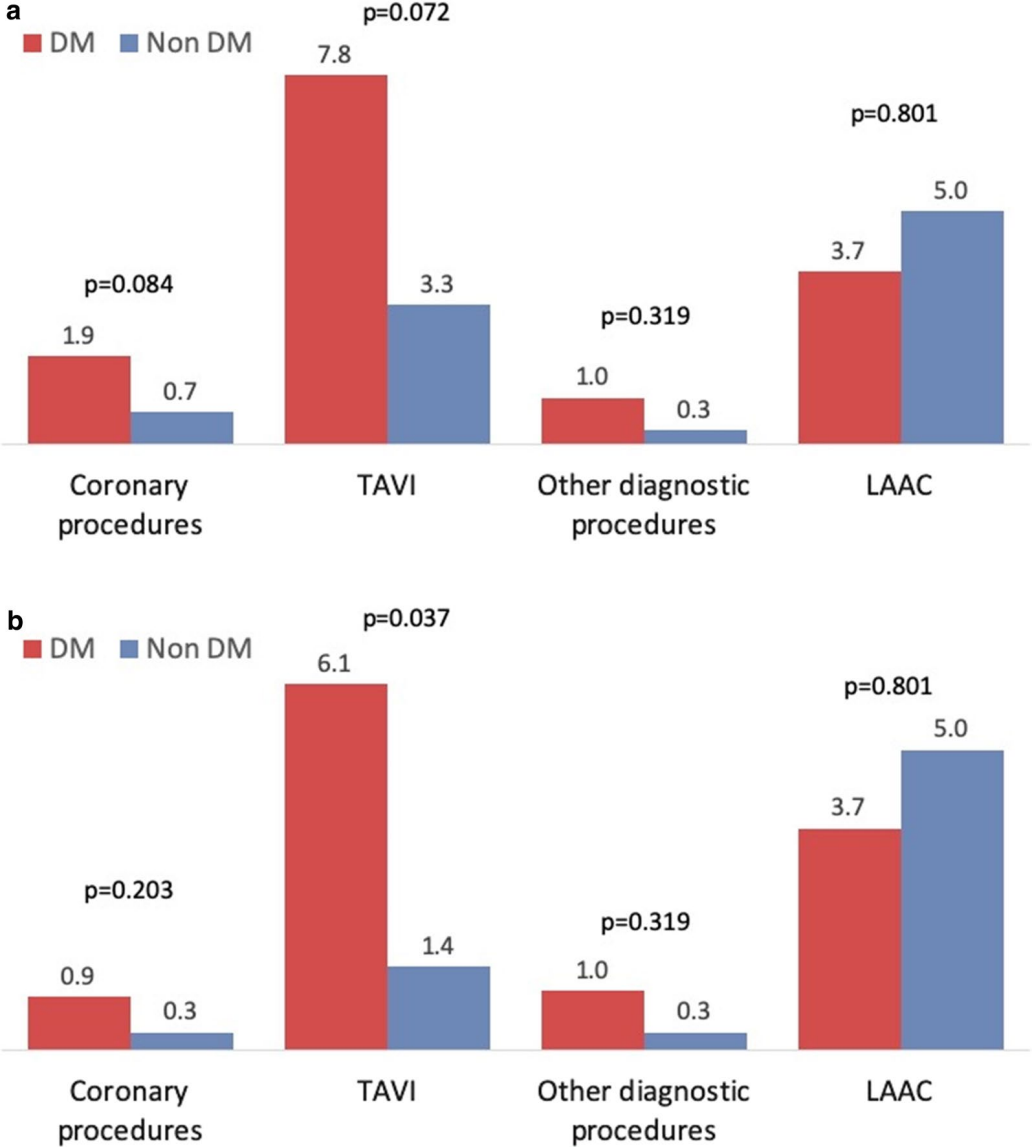
Mortality (**a**) and cardiovascular mortality (**b**) in patients with and without DM accordingly to the type of pending procedure

Figure 4 shows the mortality and cardiovascular mortality rates in patients with and without DM in the most frequent types of pending procedures. Mortality and cardiovascular mortality rates were higher in patients pending on coronary diagnostic or therapeutic procedures, TAVI, and other diagnostic procedures, but differences reached statistical significance only for cardiovascular mortality in patients pending on TAVI. In patients pending on LAAC, mortality was higher in non-DM but differences were not statistically different.

Tables 2 and 3 show the overall and cardiovascular mortality in patients with and without DM in different patient subgroups accordingly to clinical characteristics. No significant interaction was found between DM and other clinical variables (Table 4).

**Table 2:** Mortality in patients with and without DM in different patients subgroups

	Factor present					
	Yes			No		
	DM	Non-DM	P value	DM	Non-DM	P value
Age > 80 (%)	7.5	3.8	0.071	1.5	0.3	0.006
Female gender (%)	5.0	1.2	0.001	2.0	0.9	0.098
Hypertension (%)	3.3	1.1	0.003	0.0	0.7	0.362
Hypercholesterolemia (%)	3.3	1.2	0.012	1.5	0.7	0.378
Smoking (%)	1.0	0.2	0.262	3.4	1.2	0.004
Chronic renal failure (%)	9.9	2.8	0.036	2.0	0.9	0.044
Peripheral artery disease (%)	3.1	3.1	1.000	3.0	0.8	< 0.001
Previous CAD (%)	2.4	1.8	0.537	3.0	0.7	0.001
Previous infarction (%)	2.4	0.6	0.314	3.1	1.1	0.002
Previous PCI (%)	2.4	0.4	0.165	3.2	1.1	0.003
Previous CABG (%)	0.0	6.3	0.242	3.2	0.9	< 0.001
Previous valve replacement (%)	4.3	1.5	0.444	2.6	0.8	0.002
Left ventricular dysfunction (%)	1.6	0.7	0.370	3.2	1.0	0.001
NYHA > 2 (%)	7.1	2.5	0.061	2.1	0.7	0.014
CCS > 2 (%)	4.8	3.9	1.000	2.7	0.7	0.001

*PC* Percutaneous coronary intervention. *CABG* Coronary artery bypass grafting, *NYHA* New York Heart Association, *CCS* Cardiology Canadian Society

**Table 3:** Cardiovascular mortality in patients with and without DM in different patients subgroups

	Factor present					
	Yes			No		
	DM	Non-DM	P value	DM	Non-DM	P value
Age > 80 (%)	4.6	1.9	0.093	1.0	0.0	0.003
Female gender (%)	4.1	0.5	0.001	0.7	0.3	0.409
Hypertension (%)	2.3	0.5	0.002	0.0	0.2	1.000
Hypercholesterolemia (%)	2.0	0.6	0.023	1.5	0.1	0.032
Smoking (%)	0.5	0.0	0.335	2.3	0.5	0.002
Chronic renal failure (%)	7.7	1.9	0.083	1.0	0.3	0.079
Peripheral artery disease (%)	0.0	0.0	1.000	2.2	0.5	< 0.001
Previous CAD (%)	1.4	0.5	0.244	2.3	0.4	0.002
Previous infarction (%)	0.8	0.6	1.000	2.1	0.4	< 0.001
Previous PCI (%)	1.8	0.4	0.312	1.9	0.4	0.004
Previous CABG (%)	0.0	2.1	1.000	2.0	0.4	< 0.001
Previous valve replacement (%)	4.3	0.0	0.253	1.7	0.4	0.002
Left ventricular dysfunction (%)	1.1	0.7	0.635	2.2	0.4	0.001
NYHA > 2 (%)	5.1	1.2	0.049	1.6	0.2	0.004
CCS > 2 (%)	3.2	2.9	1.000	1.8	0.2	< 0.001

*PCI* Percutaneous coronary intervention, *CABG* Coronary artery bypass grafting, *NYHA* New York Heart Association, *CCS* Cardiology Canadian Society

**Table 4:** Interaction between different variables and the effect of diabetes on mortality and cardiovascular mortality

	P for interaction	
	Mortality	Cardiovascular mortality
Age > 80 (%)	0.192	0.990
Female gender (%)	0.353	0176
Hypertension (%)	0.996	0.996
Hypercholesterolemia (%)	0.774	0.375
Smoking (%)	0.799	0.994
Chronic renal failure (%)	0.499	0.790
Peripheral artery disease (%)	0.128	1.000
Previous CAD (%)	0.102	0.478
Previous infarction (%)	0.774	0.361
Previous PCI (%)	0.550	0.950
Previous CABG (%)		
Previous valve replacement (%)	0.976	0.997
Left ventricular dysfunction (%)		
NYHA > 2 (%)	0.953	0.561
CCS > 2 (%)	0.212	0.066

## Discussion

Among patients in whom an elective cardiac invasive procedure was cancelled or postponed, those with DM had a significantly higher mortality, mainly due to a higher cardiovascular mortality. Overall mortality was 3 times higher and cardiovascular mortality 4.75 times higher in DM in comparison with patients without DM.

Patients with DM had a worse clinical profile, including more advanced age, and higher prevalence of additional cardiovascular risk factors, previous cardiovascular diseases, and some co-morbidities, such as renal failure. However, DM was an independent predictor both for mortality and cardiovascular mortality, indicating that DM per se is a risk factor in this special population. The main practical implication of these findings is that elective invasive cardiac procedures should be prioritized in patients with DM. That is, during COVID-19 pandemic, invasive cardiac procedures should not be postponed in patients with DM. Even, this may be applicable not only during COVID-19 pandemic in particular [7], but also in other situations in which health care system cannot adequately attend all patients pending on invasive procedures, and in waiting list management of interventional cardiology in general.

No significant interaction was found between DM and other clinical variables. This simplifies the indication of not cancelling ICP in patients with DM, because mortality and cardiovascular mortality was higher in patients with DM irrespectively on other clinical characteristics.

The worse outcome in patients with DM may have several reasons. First, DM is associated with an impaired systolic and diastolic left ventricular function in patients without significant coronary artery disease [13], and this could have a negative impact on the outcome of patients with heart failure or valvular heart disease. Second, DM patients have a procoagulant state [14] that may increase the risk of thromboembolic events in patients pending on structural interventions. Third, the response to anti-thrombotic agents is impaired in patients with DM [15], and this could have an impact on the risk of ischemic events in patients pending on coronary interventions. Fourth, coronary artery disease is more frequent and more severe in patients with DM and severe valvular disease [16], and this could have a negative prognostic impact on clinical outcome. Finally, DM predisposes to infections [18], that constitute an important cause of non-cardiovascular death in elderly cardiovascular patients [19, 20].

DM had a negative impact on mortality in all type of pending ICP. DM is associated with more severe coronary stenosis [17], and is a very well known risk factor for mortality in patients with CAD [11, 12].

Among patients with aortic stenosis, those with DM have a higher mortality rate [21]. This may be partly explained by the higher frequency of some co-morbidities (e.g. renal insufficiency) and CAD [16], but DM also might have a direct effect on prognosis of these patients. Among patients with aortic stenosis, those with DM have a more impaired myocardial function and more severe hypertrophy [22]. In patients with aortic valve sclerosis, insulin resistance is a powerful independent predictor of subclinical left ventricular dysfunction regardless of concomitant visceral obesity and left ventricular hypertrophy [23]. Additionally, DM impairs coronary microvascular function in asymptomatic patients with severe aortic and non-obstructed coronary arteries [24]. Besides these potential explanations, cardiac mortality rate was unexpectedly high in DM patients awaiting a valvular procedure during a relatively short follow-up (45 days), and this novel finding warrants further research.

Other procedures apart from coronary and valvular interventions had also higher mortality in patients with DM. DM is a risk factor for mortality in patients on atrial fibrillation [25], and thus it is not surprising that among patients pending on LAAC, mortality was higher in diabetics.

### Study limitations

This study has several limitations. First, the main goal of this study was to evaluate the short-term consequences of delaying or postponing invasive cardiac procedures. Because of that, clinical follow-up was restricted to only 45 days. Second, metabolic control, treatment of diabetes and type of diabetes were not collected, and therefore the potential influence of these factors could not be evaluated. Finally, some patients underwent an emergent procedure due to clinical instabilization. As this occurred more frequently in patients with DM, the influence of DM on mortality in our population even may have been underestimated.

## Conclusion

Among patients in whom an elective invasive cardiac procedure is cancelled or postponed, those with DM have an especial worse clinical outcome, with higher mortality and cardiovascular mortality rates at short-term, irrespective on other clinical conditions. Elective invasive cardiac procedures should be prioritized in patients with diabetes.

## Data Availability

The datasets during and/or analysed during the current study available from the corresponding author on reasonable request.

## Abbreviations

ASD: Atrial Septal Defect
CABG: Coronary Artery Bypass Grafting
CAD: Coronary Artery Disease
CCS: Canadian Cardiac Society
COVID-19: COronaVIrus Disease 2019
DM: Diabetes
ICP: Invasive Cardiac Procedures
LAAC: Left Atrial Appendage Closure
NYHA: New York Heart Association
PCI: Percutaneous Coronary Intervention
SARS-CoV-2: Severe Acute Respiratory Syndrome CoronaVirus 2
TAVI: Transcatheter aortic valve implantation
